# Non-bracket oblique traction-hoisting construction strategy for cable-truss structures

**DOI:** 10.1016/j.heliyon.2024.e31502

**Published:** 2024-05-18

**Authors:** Mingmin Ding, Shaohua Han, Yang Wei, Yangjie Ruan, Bin Luo

**Affiliations:** a College of Civil Engineering, Nanjing Forestry University, Nanjing, 210037, China; b National Pre-stressed Engineering Center of China, Nanjing, 210096, China; c Key Laboratory of Concrete and Pre-stressed Concrete Structures of the Ministry of Education, Nanjing, 210096, China

**Keywords:** Cable-truss structure, Non-bracket oblique traction-hoisting construction strategy, Numerical simulation

## Abstract

This study describes the non-bracket oblique traction-hoisting construction strategy for cable-truss structures, which is to assemble the upper and lower radial cables, hoop cables, sling cables, and compression rods without stress at a low altitude, then hoist the cable-strut system to a high altitude by oblique traction of the upper radial cables through the jack fixed on the upper radial anchorage nodes, and finally actively tension the lower radial cables to achieve the designed shape and prestress level of the entire structure. This strategy assembles at a low altitude, requires simple operations, results in high tensioning efficiency, and does not require brackets, which could guarantee both quality and quantity in terms of completing the construction of cable-truss structures. The semilune-shaped canopy of Yueqing Stadium is constructed using this strategy. The construction simulation and disturbance stability analyses of the structure in the traction-hoisting state and prestress tensioning state are conducted using a nonlinear dynamic finite element method. In the traction-hoisting stage, the deformation changes sharply, and the hoop cables and upper radial cables make up the primary bearing substructure, while the lower radial cables are in a suspended hanging state. In the forming process, the forces of the radial and hoop cables increase gradually, and the structure finally reaches the designed state. For cable-trusses with crossed upper and lower radial cables, the additional stabilizing tooling ropes should be tied at the top of the middle rods to ensure geometric stability because they are susceptible to excessive out-of-plane displacement or even overturning, which is the least desirable at the beginning of traction hoisting.

**Table:** List of symbols

*C*	Rayleigh damping matrix.
*C*_n_	Adjustment coefficient of the maximum number of dynamic iterations.
*C*_ts_	Adjustment coefficient of the time step size.
*E*_*k*_	Total kinetic energy of the *k*th time step in dynamic analysis.
*E*_p_	Peak value of the total kinetic energy in dynamic analysis.
***F***(*t*)	Load duration vector of a component.
***K***	Stiffness matrix of a component.
***M***	Mass matrix of a component.
*N*_*ei*,0_	Initial maximum number of dynamic iterations.
*N*_*ei*,m_	Maximum number of dynamic iterations after *m*th (*m* = 1,2,3, …) updating.
*T*_0_	Initial value of the time step.
*T*_s,p_	Time step corresponding to the *E*_p_ in dynamic analysis.
***U***	Displacement vector of a component.
$\dot{U}$	Speed vector of a component.
${\dot{U}}_{k}$	Velocity vector of the *k*th time step.
***Ü***	Acceleration vector of a component.
*α*	Rayleigh damping coefficient.
*β*	Rayleigh damping coefficient.
*ω*_*i*_	Self-resonant circular frequency at step *i*.
*ω*_*j*_	Self-resonant circular frequency at step *j*.
*ξ*_*i*_	Damping ratio that corresponds to *ω*_*i*_.
*ξ*_*j*_	Damping ratio that corresponds to *ω*_*j*_.
Δ*T*_s,0_	Initial value of time step size.
Δ*T*_s,x_	Time step size after *x*th (*x* = 1,2,3, …) updating.

## 1 Introduction

A cable truss [1] is a double-layer suspension system that uses a series of convex stabilizing cables and concave load-bearing cables to replace the upper and lower radial components, which are connected through middle rods. By tensioning either the load-bearing cables (lower radial cables) or stabilizing cables (upper radial cables), or both, the cables and rods become rigid, and the whole structure takes on a stable shape and bearing capacity to resist external loads. Unlike traditional steel structures [2,3], double-layer cable nets [4], truss strings [5] or cable-strut structures [6], the upper stabilizing cables and lower load-bearing cables cut across each other near the midpoint of the projected length of some pieces. In this case, several middle rods bear compressive forces to maintain a sufficient separation distance between the upper and lower radial cables, while others bear tensile forces to connect the upper and lower radial cables as a whole. Therefore, the constructional simulation of cable trusses has a bearing on both the structural deformation and the prestress level, which needs further research.

Many studies have been carried out on the construction strategy of cable-supported structures. Li et al. [7] used an electrohydraulic proportional control system to lift a 6075 t roof frame to a height of 26.5 m. Guo et al. [8]presented the operations of hoisting, installation, and building prestress for a cable-supported latticed shell. In the research of Wang et al. [9], the temporary support frame and segment hoisting high-altitude splicing construction method was introduced for the construction of the Chiping gymnasium suspend-dome structure. Qiao et al. [10]presented a 5-step 2-phase method to assemble a cable-supported ribbed beam composite slab structure. Ding et al. [11] proposed the accumulative traction-hoisting strategy to realize the deployable construction of cable domes in the aerial suspended state. More recently, Chen et al. [12]proposed the single-side accumulative jacking construction method for large-span arched latticed shells. This method gradually jacks and assembles the shell segments until the whole structure is jacked to the final position and entirely formed, which saves construction costs, shortens the construction period, and enlarges the application area when compared with their previous studies in Shen and Luo [13].

In general, the traditional installation and tensioning of a cable-supported structure can be divided into four steps: 1) assemble the basic cable-strut network on site; 2) hoist the upper radial cables and middle rods to the designed position by tooling cables and windlasses; 3) tow and hoist the lower radial cables and hoop cables to a high altitude and fix them to the upper cable-strut system; and 4) tension the lower radial cables to the design prestress. However, existing constructional studies mainly focus on fully enclosed structures, such as circular, elliptical, and circular domes. This type of structure can be constructed symmetrically and synchronously, effectively reducing or even avoiding excessive local deformation, overall rotation of the structure, and uneven force distribution of components under asymmetric loads. In contrast to rigid structures [[14], [15], [16]], to meet the overall traction and hoisting construction requirements of semienclosed cable-supported structures, e.g., a semilune, a more detailed plan is needed to maintain a reasonable linearity of the cable-strut system, to establish the different prestress levels of different pieces, to maintain a relatively low stress of the components and to avoid the mutual disturbance between adjacent pieces in the tensioning process.

Currently, the shape-finding analysis of cable-supported structures in the construction process mostly focuses on conventional planar, folded, circular, elliptical or parallel shapes, and many theories and methods have been proposed, e.g., a dynamic relaxation method by Motro et al. [17], a force-density method by Schek [18], a nonlinear force method by Luo and Shen [19], a nonlinear force density method by Koohestani [20], a nonlinear dynamic finite element method by Ding et al. [21], a double singular value decomposition method without changing the predefined shape by Zhou et al. [22], a catenary equation-based component force balancing method by Jiang et al. [23], and a novel machine learning approach by Du et al. [24]. By solving the noncompatibility problem between cable and beam elements, Nie et al. [25] coupled the cable network and supporting frame to accurately model the real equilibrium state in an optimization model and derived the linear form for the determination of the free node coordinates from systematic equilibrium equations. Zhang et al. [26] presented a force density sensitivity form-finding design method to transform the form-finding process into a sequential quadratic programming problem. By formulating the governing equation for cable-strut structures and applying feedback control and using a proportional–integral–derivative (PID) controller to solve for the feasible prestresses based on the condition of zero structural displacements, a control method to search for feasible prestresses of cable-strut structures was proposed by Zhao et al. [27] Ma et al. [28] focused on the minimal mass design of a new cable truss structure to support concentrated vertical loads within a given span, considering both loading and unloading states. Wang et al. [29] proposed a combined digital twin (DT) and hierarchical deep learning (DL) approach for intelligent damage identification in cable dome structures, which could accurately reflect the mechanical state of actual engineering cases. Wu et al. [30] suggested two strategies named the mode compensation strategy (MCS) and mode optimization strategy (MOS) and then proposed an improved contribution-mode-based method (ICMM) to express the displacements of a real cable-strut tensile structure under a specific load. Zhu et al. [31] established a general machine learning-aided computational framework for conducting a reliable force finding process of cable domes to improve the analysis efficiency. Chen et al. [12] used an effective metaheuristic algorithm to solve the challenging problem of automatic shape design of folding origami inlays with unequal angle facets. Among these methods, the nonlinear dynamic finite element method transforms the dynamic construction process into several static analysis cases, and the whole structure is updated after each convergent static case is solved, which leads to high efficiency in simulating the large deformation of a cable-supported structure during integral tow-hoisting construction.

Considering the above aspects, this study presents a non-bracket oblique traction-hoisting construction strategy, which can be adopted to achieve installation and tensioning construction for semienclosed cable-truss structures. Then, a detailed introduction of the nonlinear dynamic finite element method is conducted to simulate the detailed process of the non-bracket oblique traction-hoisting construction strategy. Afterwards, Yueqing Stadium's semilune-shaped cable-truss roof is used as an engineering example to verify the feasibility of the construction strategy and simulation theory.

## 2 Non-bracket oblique traction-hoisting construction strategy

**Fig. 1:**
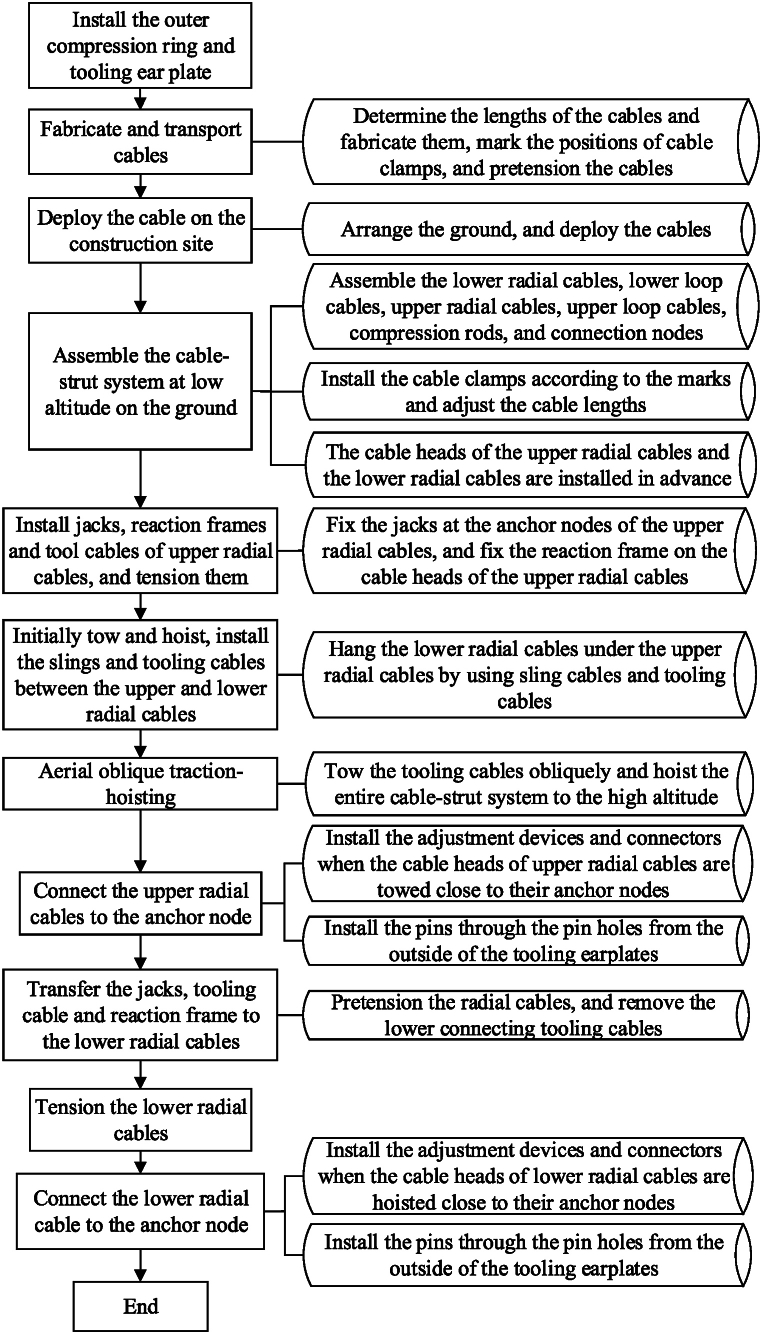
Non-bracket oblique traction-hoisting construction strategy.

The non-bracket oblique traction-hoisting strategy of constructing cable-truss structures consists of three stages: low-altitude assembly and connection, aerial hoisting and oblique traction, and high-altitude tensioning and forming. The key strategies include 1) installing the outer compression ring at the designed position, assembling the entire cable-strut system to be hoisted near the ground (including the upper radial cables, lower radial cables, suspension cables, pressure struts, and ring cables). 2) connecting the hoisting jack to the temporary trunnion plate on the outer compression ring with hanging brackets, using the tooling cables to hoist the outer heads of upper radial cables diagonally, and hoisting the entire cable-strut system to a high altitude until the upper radial cables are connected to the outer compression ring. 3) synchronously tension the lower radial cables to automatically form the structure. The specific construction steps are shown in Fig. 1.

### 2.1 Low-altitude assembly and connection

**Fig. 2:**
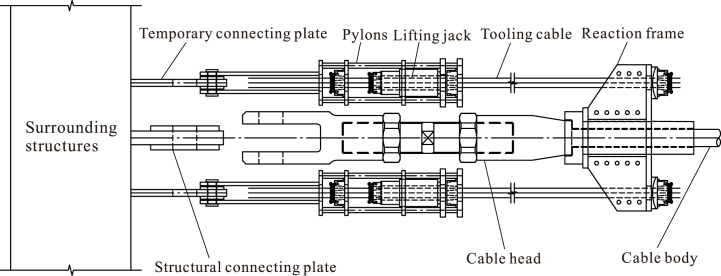
Lifting jack of cable truss.

In order to accomplish the assembly and hoisting processes on the construction site, an equipment is introduced in this study, which is illustrated in Fig. 2, which is mainly made up of two lifting jacks, two tooling cables, one reaction frame, and two temporary connecting plates. During the low-altitude assembly and connecting process, a set of equipment is set at the outer side of upper cable. By tensioning the tooling cables, the tensioning force built by lifting jacks is transferred to the surrounding structures, and the cable head of upper cable moves slowly towards the support. Finally, the cable head is connected with the connecting plate of surrounding cables, and the equipment is removed.

The sequence of low-altitude assembly and connection is as follows.

**Fig. 3:**
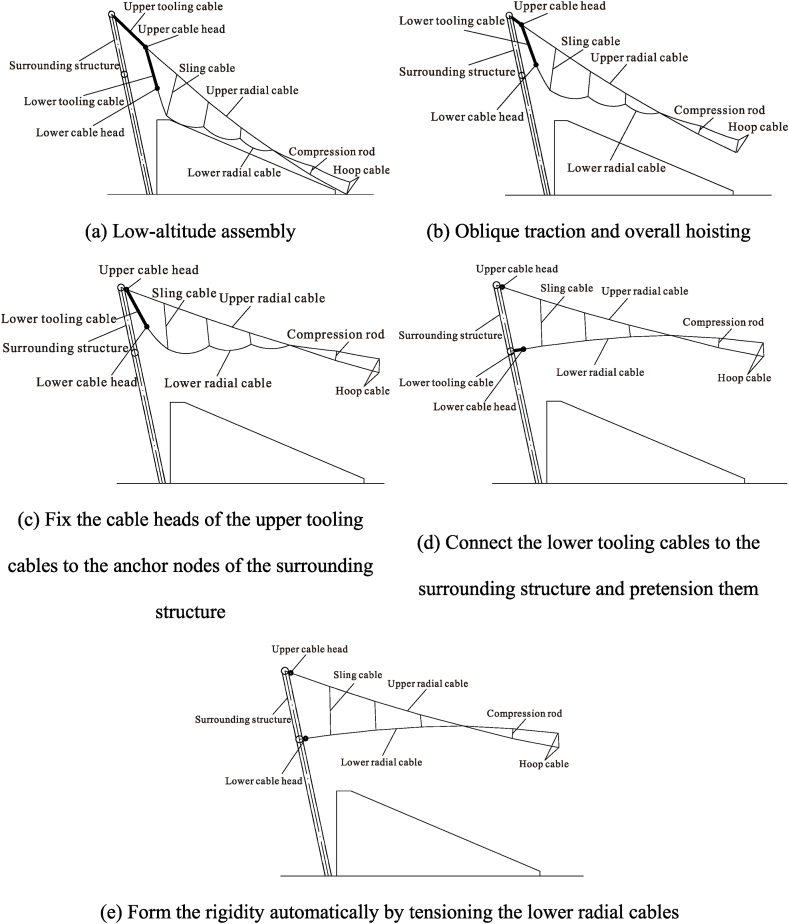
Detailed process of the non-bracket oblique traction-hoisting construction strategy.

1) Install the outer compression ring at the design position.
2) Assemble upper radial cables, lower radial cables, upper hoop cables, lower hoop cables, and middle rods (including sling cables and compression rods) under an unstressed state at the low-altitude position below the cable truss.
3) Fix and install the jacks for traction hoisting on the upper radial anchor nodes, install a reaction frame on the side heads of the upper radial cables, install the tooling cables for oblique traction between the jacks and reaction frame, and pretension them, as shown in
4) Gradually raise the upper radial cables off the ground by a certain height during the initial operating phase of traction hoisting.
5) Install the tooling cables between the cable heads of the upper and lower radial cables and hang the lower radial cables' heads onto the upper radial cables' heads.
6) Install the sling cables between the upper and lower radial cables, as shown in Fig. 3a.

### 2.2 Aerial oblique traction hoisting

The following is the working procedure of aerial oblique traction hoisting.

1) Start the jacks, fixed to the upper radial anchor nodes, and raise the entire cable-strut system to a high altitude by diagonally towing, as shown in Fig. 3b.
2) Connect the cable heads of the upper radial cables to the upper radial anchor nodes of the surrounding structure and remove the jacks, reaction frame, and tooling cables, as shown in Fig. 3c.

### 2.3 High-altitude tensioning and forming

The sequence of high-altitude tensioning and forming is as follows.

1) As shown in Fig. 3d, install the tensioning jack on the lower radial cables' anchor nodes, install the reaction frames on the lower radial cables' heads, install and pretension the tooling cables between the jacks and reaction frames, and finally, remove these tooling cables.
2) Fix the jacks on the lower radial cable anchor nodes, simultaneously tensioning the tooling cables of the lower radial cables, attaching the lower radial cable heads to the lower radial cable anchor nodes, and tensioning the entire structure to generate the overall stiffness, as shown in Fig. 3e.

### 2.4 Primary objectives

In order to reduce the extensive high-altitude work and duration of bracket assembly, which are needful in conventional methods (i.e. all-round scaffold method and high-altitude spelling method), the non-bracket oblique traction-hoisting construction strategy is introduced in this study. In theory, the low-altitude assembly and aerial traction hoisting avoid the erection of many temporary supports and greatly decrease construction costs, improve the assembly quality, and reduce the construction period, under the premise of clear and controllable structural deformation and stress. In this case, the primary objectives of using non-bracket oblique traction-hoisting construction strategy are: 1) Track and simulate the structural form and internal forces of components throughout the entire construction process; 2) Ensure reasonable internal forces of components and the overall stability of the structure during construction.

### 2.5 Strategy features

**Fig. 4:**
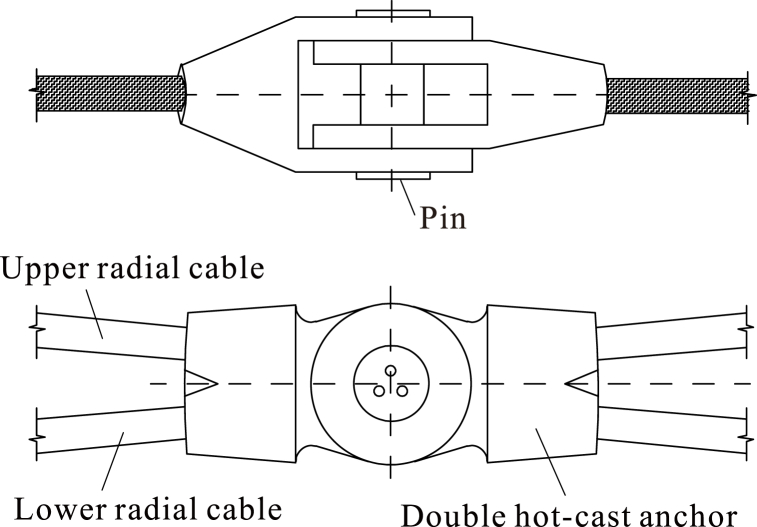
Intersection joints connecting upper and lower radial cables.

1) If the unstressed lengths of all the cables are known, the formed shape and prestressed state can also be determined. Therefore, the passively tensioned cable-strut system should be assembled according to certain unstressed lengths. While inputting prestress, the active cables are tensioned to build the prestress of the whole structure. In this case, the actively tensioned cables are the key components. Radical cables should be selected as the active tensioned cables for radially distributed cable trusses to ensure tensioning quality. Furthermore, the internal forces of the lower radial cables are smaller than those of the upper radial cables. To reduce the tensioning tonnage, the lower radial cables should be chosen as the actively tensioned cable.
2) The upper and lower radial cables are disconnected by setting double hot-cast anchor heads at the intersection joints, as shown in Fig. 4. This arrangement avoids sliding of cable bodies and constrains the upper and lower radial cables to the same vertical plane, which ensures the clarity of the force transmission and the reliability of the connection.

3) Due to the low scaffolding usage and the good construction efficiency in setting the cables and rods to the designate location, and simultaneously establishing the stiffness of the overall structure through tensioning by programmable logic controller hydraulic system (PLC) afterwards, the non-bracket oblique traction-hoisting construction strategy is almost applicable to any environment where machinery can operate. As for the limitations of the construction site, a manually leveled site is sufficient, as stated in Luo et al. [32] and Ding et al. [33].

### 2.6 Principles

The small hoisting forces, the few high-altitude works, and the high efficiency of tensioning make non-bracket oblique traction-hoisting construction strategy easy to realize. However, to guarantee the reliability and safety of construction, the following principles must be complied.

1. The upper radial cables keep the shape of “ω” in the early stages of construction to fulfill the stability of the whole structure.
2. The maximum tensile force of a cable must be no more than 50 % of its bearing capacity.
3. The maximum compressive force of the compression rods and surround steel structures must be no more than 50 % of its vertical buckling load (GB 50017-2017) [34]according to Eq. (1).

(1) $$\frac{N}{\varphi A_{s}f}\leq 1.0$$

where *N* is the vertical buckling load, *φ* is the stability factor, *A*_*s*_ is the sectional area of a steel component, and *f* is the design value of tensile strength.

## 3 The improved nonlinear dynamic finite element method (improved NDFEM)

### 3.1 General idea

Because large deformation in joints and force variations in cables occur during the non-bracket oblique traction-hoisting stages, the whole construction procedure is a dynamic process. To meet the requirements of both calculation efficiency and calculation accuracy, the construction procedure could be divided into a certain number of static cases. In this case, the whole analysis of the nonlinear dynamic finite element model [24] includes the following steps.

1) Establish a numerical model that conforms to the structural state before tensioning.
2) Divide the construction procedure into several static cases.
3) Give the tensioned cables initial strains to simulate the shortening lengths between Case #0 and Case #1.
4) Conduct dynamic equilibrium iterations and update the model when the total kinetic energy reaches its peak.
5) Perform nonlinear static analysis and update the structural model to verify the static equilibrium state. If the static analysis converges, then Case #1 is completed.
6) Take the updated model in step 5) as the initial model, and repeat steps 3) to 5) to complete Case #2.
7) Repeat step 6) to complete Cases #3, #4, …, until the whole construction procedure is finished.

**Fig. 5:**
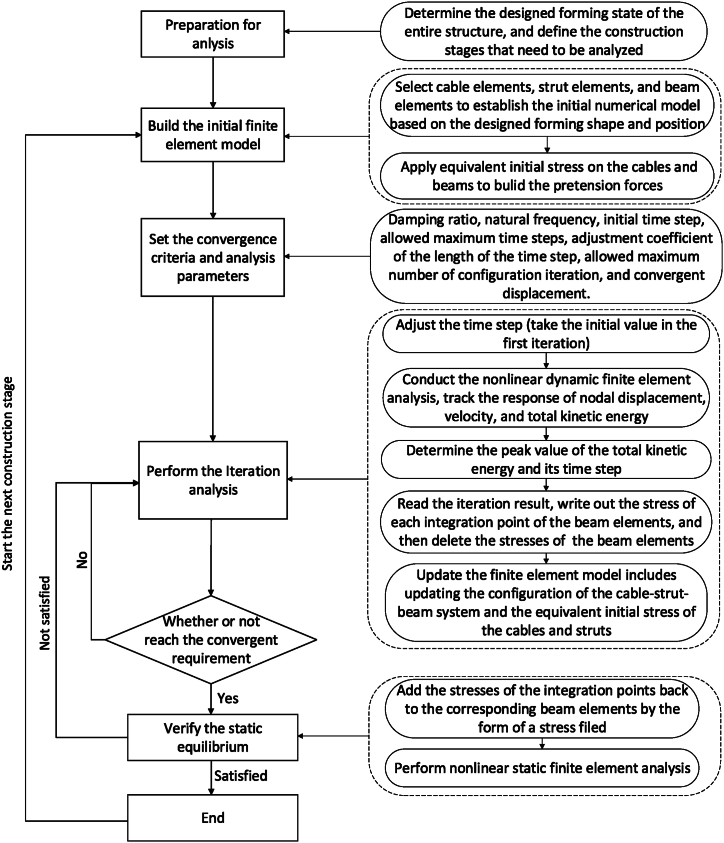
Detailed operation process of modified NDFEM.

The detailed process is illustrated in Fig. 5.

### 3.2 Key operations

Between each pair of static cases, the classical dynamic balance equations, e.g., Eqs. (2), (3)), and the Rayleigh damping matrices [35], e.g., Eqs. (4), (5)), are adopted in the nonlinear dynamic analysis to calculate the variations in the internal force and nodal deformation of each component.

(2) $$M\ddot{U}+C\dot{U}+KU=F\left(t\right)$$

(3) C=αM+βK

(4) $$\alpha =\frac{2\omega_{i}\omega_{j}\left(\xi_{i}\omega_{j}-\xi_{j}\omega_{i}\right)}{\omega_{j}^{2}-\omega_{i}^{2}}$$

(5) $$\beta =\frac{2\left(\xi_{j}\omega_{j}-\xi_{i}\omega_{i}\right)}{\omega_{j}^{2}-\omega_{i}^{2}}$$

If *ξ*_*i*_ = *ξ*_*j*_ = *ξ*, Eqs. (4), (5)) can be simplified to Eqs. (6), (7)).

(6) $$\alpha =\frac{2\omega_{i}\omega_{j}\xi }{\omega_{j}+\omega_{i}}$$

(7) $$\beta =\frac{2\xi }{\omega_{j}+\omega_{i}}$$

After performing the *n*th (*n* = 1,2,3, …*N*_*ei*,m-1_) dynamic equilibrium iteration, the total kinetic energy is calculated through Eq. (8), and the follow-up operations are performed according to the following steps.

**Fig. 6:**
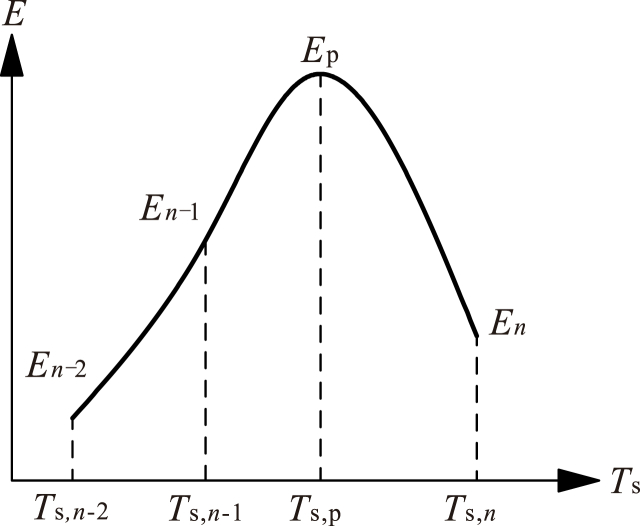
Peak value of total kinetic energy and its time step.

a) If *E*_*n*_ > *E*_*n*−1_ and *n* < *N*_ei,*m*-1_, continue this dynamic analysis and start the (*n*+1)th time step.
b) Otherwise, if *E*_*n*_ > *E*_*n*−1_ and *n* = *N*_ei,*m*-1_, update the numerical model according to the total displacement of the *n*th dynamic equilibrium iteration, adjust the maximum number of dynamic iterations as *N*_ei,*m*_ = *N*_ei,*m-*1_ × *C*_n_, and restart the dynamic analysis.
c) Otherwise, if the analysis does not converge, adjust the initial time step size as Δ*T*_s,x_ = Δ*T*_s,x-1_ × *C*_ts_, and restart the dynamic analysis.
d) Otherwise, if *E*_*n*_ < *E*_*n*−1_ and *n* < *N*_ei,*m*-1_, find the peak value *E*_p_ and its time step *T*_s,p_ from Fig. 6, and find the total displacement of this time step by linear interpolation. Then, find the next static equilibrium state and renew the whole model.

(8) $$E_{k}=\frac{1}{2}{\dot{U}}_{k}^{T}M{\dot{U}}_{k}$$

### 3.3 Improvement of model updates

The NDFEM can establish nonlinear finite element motion equations for all cable nodes to simulate the form and structural response of each construction stage, making it suitable for the construction simulation of cable nets. Nevertheless, unlike traditional cable nets, the compressive rods and surrounding structure of cable trusses are circular steel pipes, and their deformations are affected by bending stress during construction. In this case, it is necessary to introduce a beam element that can transfer both bending stress and axial force to simulate the response of these steel pipes and add the iteration principle of beam elements in NDFEM to make the construction simulation of cable truss more accurate.

The model update includes three parts, namely, configuration update, internal force update, and component length update. After updating the coordinates of all nodes with the improved NDFEM, the component length of the cable truss subsequently changes.

A cable truss is mainly composed of three types of elements: cable components, strut components, and beam components. For cable elements that need to maintain their length values (i.e., radial cables, hoop cables, and sling cables), adjust the equivalent initial stress of these elements during each iteration to maintain their lengths. For cable elements that need to maintain their internal force values (i.e., tooling cables), the equivalent initial stress of these elements is not changed after each iteration. For beam elements (i.e., compression rods, surrounding structural members) whose length values need to be maintained, the stress at each integration point is written before each iteration, then the equivalent initial stresses of these elements are adjusted to maintain the length of these elements, and finally, the stresses of the integration points are added back to the corresponding elements after each iteration. All the bottom endpoints of the columns of the surrounding structure is set as hinged.

## 4 Calculation and results of an example

### 4.1 Project overview

**Fig. 7:**
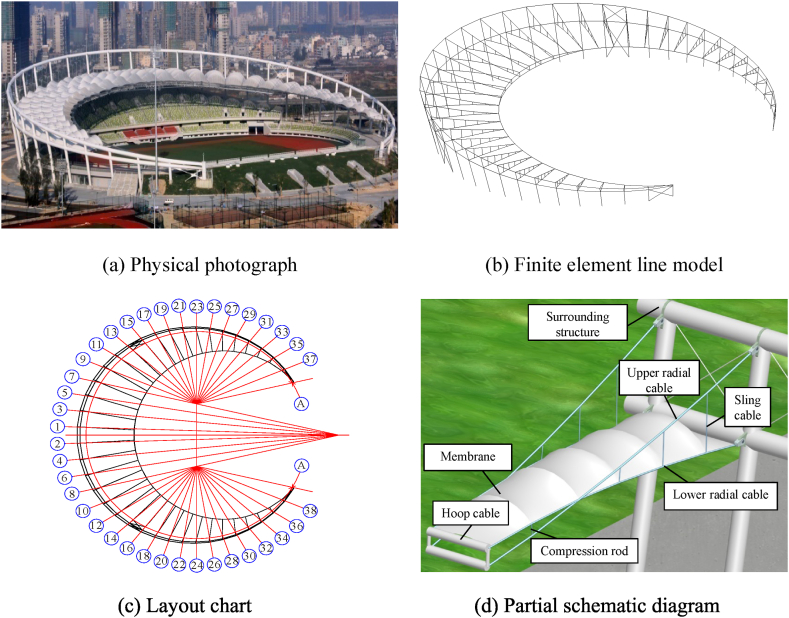
Physical photograph, FEM and partial schematic diagram of the semilune-shaped nonclosed cable-truss structure.

As shown in Fig. 7(a–d), the canopy of Yueqing Stadium [36] in Zhejiang Province is a semilune-shaped nonclosed cable-truss structure with a length of approximately 229 m from north to south and a width of approximately 211 m from east to west.

**Fig. 8:**
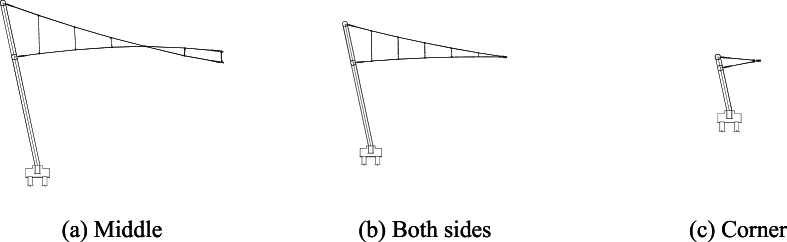
Sections of cable trusses.

The upper radial cables, lower radial cables, hoop cables, and middle rods make up the primary structure of each cable truss, which is a typical double-layered suspension cable structure. With hoop cables connecting the ends, 38 cable trusses are arranged radially. The suspension end has a maximum elevation of +26.590 m, a maximum length of 440 m, and a maximum suspension of approximately 57 m (specific details are shown in Fig. 8).

As shown in Fig. 8a, the upper and lower radial cables of the 8 cable trusses located in the central region are crossed and linked with sling cables and compression rods. In Fig. 8b, only sling cables connect the upper and lower radial cables of the 24 cable trusses on the side. The remaining 6 cable trusses at the corners are not connected, as displayed in Fig. 8c.

The roof substructure is a two-hinged steel arch with a circumferential tectonic cable to balance the horizontal thrust of the steel arch, which is covered with a PTFE membrane [37]. The perimeter is a tapered grid structure formed by upper and lower ring steel beams (1500 mm in diameter and 50 mm in wall thickness) and inclined columns (1300 mm in diameter and 40 mm in wall thickness) injected with C50 concrete, with a maximum elevation of +42.000 m at the top of the inclined columns. Cross-cables are installed between the local inclined columns.

Table 1 shows the project's materials and specifications, and the pressure struts are round steel pipes (diameter 152 mm, wall thickness 10 mm) as the same as the final design documents of the design institute, calculated by the bearing capacity limit state. The Galfan-coated steel strand ropes are made of high-strength steel wires with a round cross-section that have been twisted together, with a mixed rare earth alloy coating of 95 % zinc +5 % aluminium.

**Table 1:** Cable materials and specifications.

Tensile cables			Type	Tensile strength /MPa	Diameter /mm
Cable-truss structure	Radial cable	Piece 1∼36	Galfan-coated stranded wire cable	1,670	100
		Piece 37∼38	Galvanized steel tie rod	650	105
	Hoop cable		Galfan-coated stranded wire cable	1,670	8 × 125
	Sling cable		Galfan-coated stranded wire cable	1,670	38
Structural cross-cables between diagonal columns			Galvanized steel tie rod	650	150
Substructure membrane surfaces	Structural cable		Galfan-coated stranded wire cable	1,670	32

The prestress of the entire structure is built by adding initial strains to the cables, as shown in Eq. (9).

(9) $$P=E\varepsilon A_{c}$$

where *P*, *ε*, *E*, and *A*_*c*_ denote the initial pretension force, the initial strain, the elastic modulus, and the sectional area of a cable, respectively.

### 4.2 Numerical calculation model

**Fig. 9:**
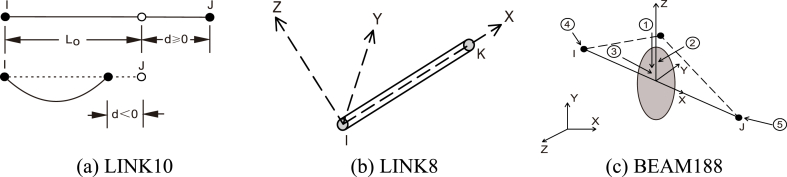
Element types used in numerical simulation.

The numerical calculation model is constructed by drawing line components in AutoCAD, and then the nonlinear finite element analysis program ANSYS is used to import the line model, model the cable force, nodal displacement, and component stress of the whole structure. A three-dimensional two-node cable element (LINK10), shown in Fig. 9a–is used to simulate the flexible radial cables and hoop cables. A three-dimensional two-node spar element (LINK8), illustrated in Fig. 9b–is used to simulate the middle sling cables. A three-dimensional two-node beam element (BEAM188), present in Fig. 9c–is utilized to simulate the middle compression rods and surrounding structural members. Accomplish perfect bonds are created between adjacent components by sharing the same node and the same coordinates. The prestress of the entire structure is built by adding initial strains to the cables, as shown in Eq. (9), and the initial pretension force of all the pieces is listed in Table 2, in which the piece number follows Fig. 7c.

(9) $$P=E\varepsilon A_{c}$$

**Table 2:** Initial pretension forces of the numerical calculation model.

Piece No.	Radial cable/kN		Hoop cable/kN		Piece No.	Radial cable/kN		Hoop cable/kN	
	Upper	Lower	Upper	Lower		Upper	Lower	Upper	Lower
1	1,677.1	1,344.2	13761,000	10076,000	20	1,323.3	1,275.1	23998,000	
2	1,677.1	1,344.2	13761,000	10076,000	21	1,346.4	1,266.2	24011,000	
3	1,672.2	1,329	13779,000	10076,000	22	1,346.4	1,266.2	24011,000	
4	1,672.2	1,329	13779,000	10076,000	23	1,380.2	1,282.5	24022,000	
5	1,637.8	1,363.4	13808,000	10075,000	24	1,380.2	1,282.5	24022,000	
6	1,637.8	1,363.4	13808,000	10075,000	25	1,424.6	1,322.6	24031,000	
7	1,723	1,318.5	13845,000	10074,000	26	1,424.6	1,322.6	24031,000	
8	1,723	1,318.5	13845,000	10074,000	27	1,489.3	1,382	24036,000	
9	1,587.1	1,367	23907,000		28	1,489.3	1,382	24036,000	
10	1,587.1	1,367	23907,000		29	1,578.9	1,466.4	24038,000	
11	1,600.7	1,259.5	23928,000		30	1,578.9	1,466.4	24038,000	
12	1,600.7	1,259.5	23928,000		31	1,690.6	1,577.1	24035,000	
13	1,367.9	1,365.8	23947,000		32	1,690.6	1,577.1	24035,000	
14	1,367.9	1,365.8	23947,000		33	1,820.5	1,724.8	24025,000	
15	1,369.2	1,298.7	23965,000		34	1,820.5	1,724.8	24025,000	
16	1,369.2	1,298.7	23965,000		35	2,001.3	1,888.2	24015,000	
17	1,350.4	1,269.9	23982,000		36	2,001.3	1,888.2	24015,000	
18	1,350.4	1,269.9	23982,000		37	2,158.3	2,018.3	23988,000	
19	1,323.3	1,275.1	23998,000		38	2,158.3	2,018.3	23988,000	

*Because pieces 9–38 only have one hoop cable each, as illustrated in Fig. 8b, the “hoop cable” lines of these pieces are merged.

### 4.3 Construction analysis

Based on the non-bracket oblique traction-hoisting strategy and the nonlinear dynamic finite element method, tooling cables are set up, and the outer compression ring is taken as the support to simulate the construction progress in ANSYS. The analysed static cases are shown in Table 3, and the 100 % lengths of the tooling cables are displayed in Table 4.

**Table 3:** Analysed static cases during the tow-hoisting and tensioning processes.

Construction Phase	Case No.	The lengths of the upper tooling cables	The lengths of the lower tooling cables	The degree of adjustability for the outmost lower radial cables/mm
Traction-hoisting	1	100 %	100 %	+250
	2	80 %	100 %	+250
	3	60 %	100 %	+250
	4	40 %	100 %	+250
	5	20 %	100 %	+250
	6	0 %	100 %	+250
Tensioning	7	–	–	+250
	8	–	–	+200
	9	–	–	+150
	10	–	–	+100
	11	–	–	+50
Rigid forming	12	–	–	±0

**Table 4:** 100 % lengths of the tooling cables.

Piece No.	The lengths of the upper tooling cables/mm	The lengths of the lower tooling cables/mm	Piece No.	The lengths of the upper tooling cables/mm	The lengths of the lower tooling cables/mm
1	9,372	9,984	20	11,449	7,078
2	9,372	9,984	21	11,449	7,561
3	9,372	9,984	22	11,449	7,561
4	9,372	9,984	23	11,451	5,900
5	9,372	9,904	24	11,451	5,900
6	9,372	9,904	25	11,452	5,192
7	9,372	9,772	26	11,452	5,192
8	9,372	9,772	27	11,876	4,959
9	9,372	9,375	28	11,876	4,959
10	9,372	9,375	29	11,876	4,883
11	9,374	9,066	30	11,876	4,883
12	9,374	9,066	31	11,878	4,857
13	10,410	8,539	32	11,878	4,857
14	10,410	8,539	33	11,880	4,840
15	10,411	8,232	34	11,880	4,840
16	10,411	8,232	35	12,054	4,829
17	10,413	7,577	36	12,054	4,829
18	10,413	7,577	37	12,056	4,822
19	11,449	7,078	38	12,056	4,822

To reduce the total time and ensure the accuracy of the analysis, the analysis is carried out sequentially from Case #12 to Case #1 in the inverse construction sequence, using the design forming state as the initial model. After adding tooling cables and adjusting their original lengths, the previously converged whole model is used as the initial model for the analysis of the following static case. Furthermore, to guarantee that the dynamic equilibrium iterations reach the peak of total kinetic energy and each static analysis can reach convergence, one static case is divided into 1000 segments by equally divide the time step size.

**Fig. 10:**
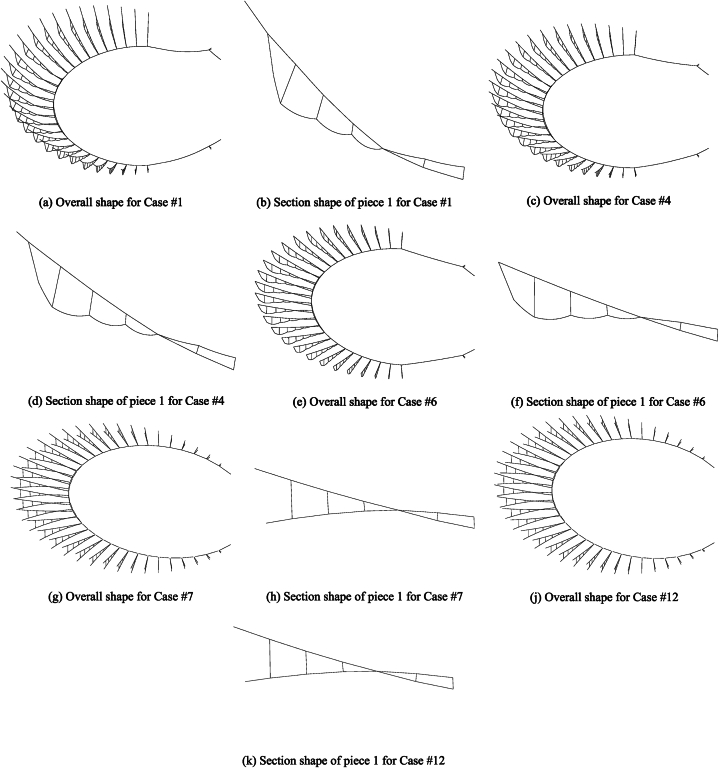
Configurations of the construction cases in the static equilibrium state.

**Fig. 11:**
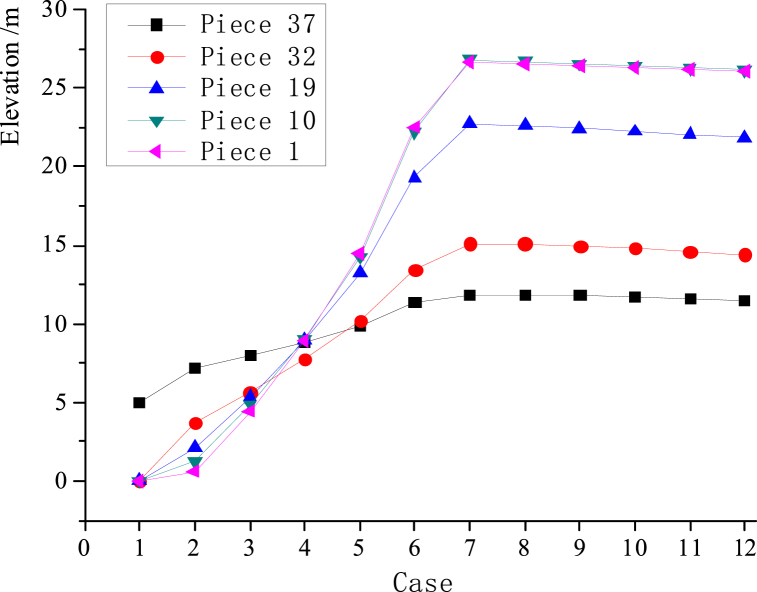
The altitude change curves of ring-cable nodes during construction.

**Fig. 12:**
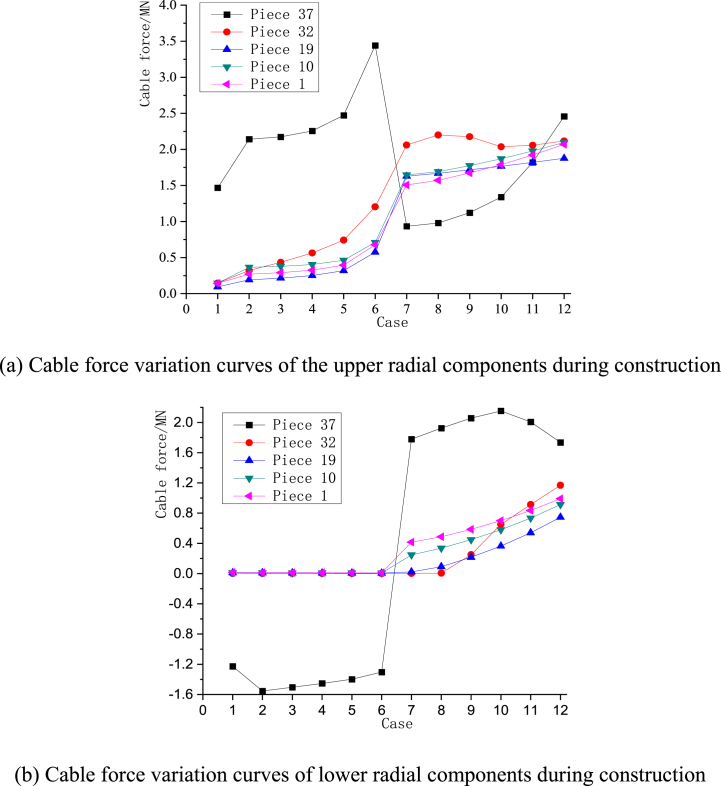
The force change curves of the upper and lower radial cables during construction.

Fig. 10(a–k) depict the static equilibrium shape under different static cases, Fig. 11 depicts the elevation change curves of the hoop cable nodes, and Fig. 12(a) and (b) depict the force change curves of the upper and lower radial cables.

The figures show that (1) in the traction-hoisting stage from Case #1 to Case #6, the structural shape changes significantly, and the upper radial cables are taut, while the lower radial cables are suspended under the upper radial cables; (2) in the tensioning stage from Case #7 to Case #12, the forces of the upper and lower radial cables gradually increase; and (3) due to the short lengths, the outermost upper and lower radial cables of pieces 37 and 38 use galvanized steel tie rods, whose internal forces change abruptly in Cases #6 and #7. The forces of the upper radial cables of piece 37 and piece 38 reach a peak (3441 kN) in Case #6 and decrease to a valley (934 kN) in Case #7. In the traction-hoisting stage, most of the lower radial cables are unstressed, while the lower radial steel rods of piece 37 and piece 38 give the maximum variations; the force reaches a pressure peak (−1555 kN) in Case #2, decreases slightly (−1303 kN) in Case #6, turns to tension (1779 kN) in Case #7, and reaches peak tension (2153 kN) in Case #10. In conclusion, the upper radial cables and the hoop cable form the main bearing substructure during the traction-hoisting stage, and the lower radial cables are suspended beneath it based on the change in shape and cable forces. Finally, the structure is stiffened and formed by tensioning the lower radial cables. Because the upper and lower radial steel tie rods of pieces 37 and 38 are installed prior to traction hoisting, their internal forces change dramatically during the construction process, exceeding the internal design forces of the final forming, and even the lower radial steel ties are subjected to significant pressure during this period.

### 4.4 Out-of-plane stability analysis

As previously stated, the lower radial cables are suspended beneath the upper radial cables during the traction-hoisting stage. The upper and lower radial cables of the eight central cable trusses are crossed in the midpoint, and the hoop cables are divided into upper and lower hoop cables here, with the lower radial cables being above the upper radial cables at the overhanging ends. Because the lower radial cables and upper hoop cables are loose and sagging during this construction stage, an out-of-plane stability analysis must be performed to prevent excessive out-of-plane displacements of the middle rods at the overhanging end, which would otherwise result in overturning instability.

A slight disturbance force is used to introduce the out-of-plane initial displacement. The specific method and the stability determination principle are as follows:

Based on the converged static models, a small horizontal annular concentrated force is applied to the upper node of each middle rod in the same rotational direction to calculate the static equilibrium state of the whole structure. If the middle rods reflect excessive out-of-plane displacements or even overturning, the construction state is unstable; otherwise, if the middle rods are in static equilibrium after a certain out-of-plane displacement, the construction state is basically stable. In the second condition, if the middle rods could return to their predisturbance state after removing the disturbance force, the construction state is stable.

Due to the suspended shape and the low cable forces without traction and tensioning, the structural state in Case #1 has the most unfavourable middle rod stability. As a result, the nonlinear dynamic finite element method is utilized to simulate the disturbance stability analysis of the whole structure in Case #1, and the disturbance force is assumed to be 1 kN.

**Fig. 13:**
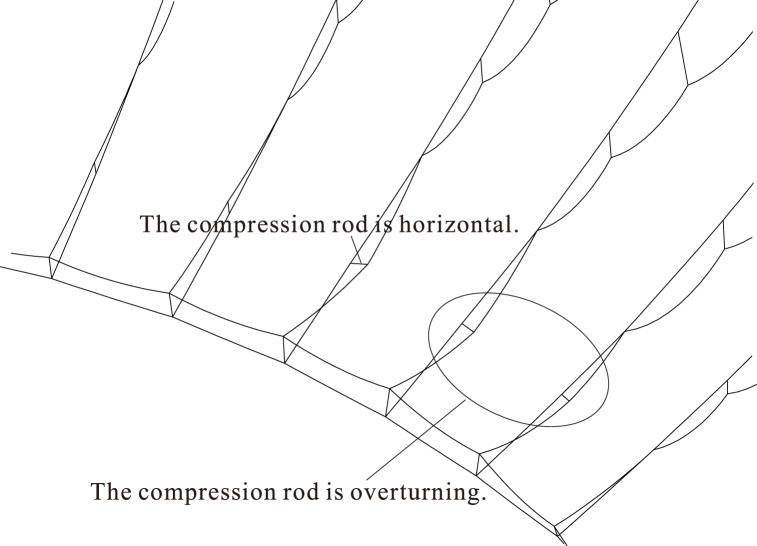
Configurations of Case #1 in the static equilibrium state with external force application and removal.

**Fig. 14:**
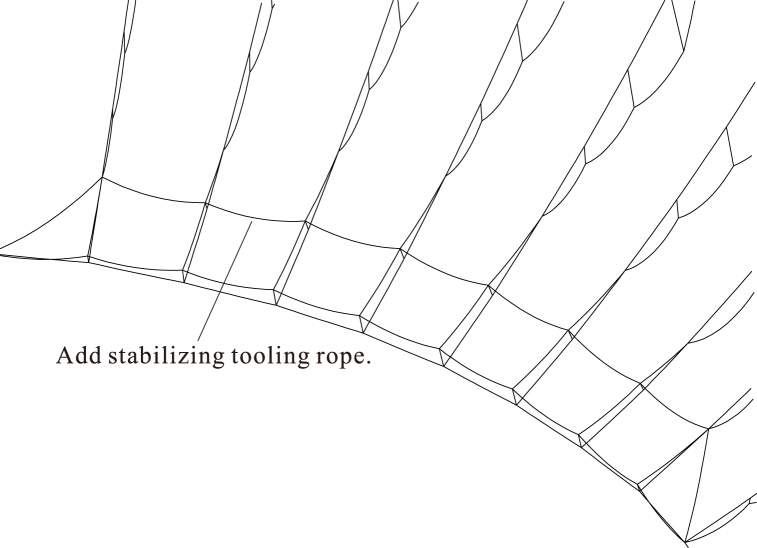
Configurations of Case #1 in the static equilibrium state to create stable cables.

Fig. 13 shows that a portion of the middle rod and lower radial cables at the end of the overhang turns over under the upper radial cable. Additional stabilizing tooling ropes are installed between the central 10 pieces of cable trusses to maintain the out-of-plane stability of the middle rods during the non-bracket oblique traction-hoisting construction. The linear distances between the top nodes of the middle rods of adjacent cable trusses after forming, i.e., the stress-free lengths, are used as the original lengths of the stabilizing tooling ropes to avoid the influence on the construction phase of tensioning. As shown in Fig. 14 and the corresponding analysis, the stabilizing tooling ropes are established in the model of Case #1, and all the middle rods are stable after reaching the static equilibrium state.

## 5 Discussion

This research introduces the non-support oblique traction-hoisting construction strategy and conducted the construction simulation of a typical example by using the improved NDFEM. The results indicate that this construction strategy reduces high-altitude work, decreases bracket erection time and cost, which provides a superior option for the actual construction scheme of cable-truss structures. Nevertheless, similar to any other construction simulation studies, the construction strategy and analysing method introduced in this study are not perfect. The results reported have certain limitations, summarized as follows.

1. The usability of the non-support oblique traction-hoisting construction strategy, currently based on the semilune-shaped cable-net canopy of Yueqing Stadium, needs to be tested and validated in further work to expand its applicability.
2. Compared to existing NDFEM researchs [21,32,36], the improved NDFEM introduced in this study adds the simulation function of bending stress for beam elements, by output and input the stress at each integration point of beam elements before and after model's updating. However, the compression rods of cable-trusses with both ends hinged mainly subjected to axial force, only initial bending or defect leads to small bending stress. Furthermore, due to the large stiffness, the surrounding structures are usually simplified and only provide fixed hinged ends for cable-trusses. In this case, the simulation function of beam elements could not be fully ultilized for cable-trusses. If the proposed analysis method is conducted to simulate rigid roof structures, the deformations of beam elements under complex stress will be more complicated and the iterative operations will be more difficult to reach convergence. In our subsequent studies, the improved NDFEM will be invested in the simulation of suspen-domes and reticulated shells.

## 6 Conclusion

In this study, the non-bracket oblique traction-hoisting construction strategy for cable-truss structures is proposed. This strategy involves assemble the upper and lower radial cables, hoop cables, sling cables, and compression rods without stress at a low altitude, then hoist the cable-strut system to a high altitude by oblique traction of the upper radial cables through the jack fixed on the upper radial anchorage nodes, and finally actively tension the lower radial cables to achieve the designed shape and prestress level of the entire structure. This approach reduces high-altitude work, decreases bracket erection time and cost, is more easily accomplished, and increases construction efficiency.

In conjunction with the semilune-shaped canopy of Yueqing Stadium, the structural response in traction-hoisting simulation, a tensioning and forming analysis, and an out-of-plane disturbance stability study was investigated. During the traction-hoisting stage, the upper radial cables and hoop cables form the main bearing substructure, while the lower radial cables are loosely hanging. Moreover, the forces of the upper and lower radial cables gradually increase during the tensioning process, and finally, the whole structure is formed.

Due to the existence of the middle rods and hoop cables at the central intersections of the upper and lower radial cables, the middle rods are prone to excessive out-of-plane displacement or even overturning. The analysis suggests that by installing the stabilizing tooling ropes between the top nodes of the middle rods of adjacent cable trusses, all the middle rods could maintain out-of-plane stability after reaching the static equilibrium state.

## Data availability statement

All data included in this study are available upon request by contact with the corresponding author.

## CRediT authorship contribution statement

**Mingmin Ding:** Writing – review & editing, Software, Methodology, Conceptualization. **Shaohua Han:** Writing – original draft, Data curation. **Yang Wei:** Visualization, Investigation. **Yangjie Ruan:** Validation, Software. **Bin Luo:** Supervision.

## Declaration of competing interest

The authors declare that they have no known competing financial interests or personal relationships that could have appeared to influence the work reported in this paper.
